# World Malaria Report: time to acknowledge *Plasmodium knowlesi* malaria

**DOI:** 10.1186/s12936-017-1787-y

**Published:** 2017-03-31

**Authors:** Bridget E. Barber, Giri S. Rajahram, Matthew J. Grigg, Timothy William, Nicholas M. Anstey

**Affiliations:** 1 Global and Tropical Health Division, Menzies School of Health Research and Charles Darwin University, PO Box 41096, Darwin, NT 0811 Australia; 2 Infectious Diseases Society Sabah-Menzies School of Health Research Clinical Research Unit, Queen Elizabeth Hospital, 88200 Kota Kinabalu, Sabah Malaysia; 3 Clinical Research Centre, Queen Elizabeth Hospital, 88200 Kota Kinabalu, Sabah Malaysia; 4 Jesselton Medical Centre, Jalan Metro 2, 88300 Kota Kinabalu, Sabah Malaysia

**Keywords:** *Plasmodium knowlesi*, Malaria, World Malaria Report, Malaysia

## Abstract

**Background:**

The 2016 World Health Organization (WHO) World Malaria Report documents substantial progress towards control and elimination of malaria. However, major challenges remain. In some regions of Southeast Asia, the simian parasite *Plasmodium knowlesi* has emerged as an important cause of human malaria, and the authors believe this species warrants regular inclusion in the World Malaria Report.

**Main text:**

*Plasmodium knowlesi* is the most common cause of malaria in Malaysia, and cases have also been reported in nearly all countries of Southeast Asia. Outside of Malaysia, *P. knowlesi* is frequently misdiagnosed by microscopy as *Plasmodium falciparum* or *Plasmodium vivax.* Thus, *P. knowlesi* may be underdiagnosed in affected regions and its true incidence underestimated. Acknowledgement in the World Malaria Report of the regional importance of *P. knowlesi* will facilitate efforts to improve surveillance of this emerging parasite. Furthermore, increased recognition will likely lead to improved delivery of effective treatment for this potentially fatal infection, as has occurred in Malaysia where *P. knowlesi* case-fatality rates have fallen despite rising incidence. In a number of knowlesi-endemic countries, substantial progress has been made towards the elimination of *P. vivax* and *P. falciparum*. However, efforts to eliminate these human-only species should not preclude efforts to reduce human malaria from *P. knowlesi*. The regional importance of knowlesi malaria was recognized by the WHO with its recent Evidence Review Group meeting on knowlesi malaria to address strategies for prevention and mitigation.

**Conclusion:**

The WHO World Malaria Report has an appropriate focus on falciparum and vivax malaria, the major causes of global mortality and morbidity. However, the authors hope that in future years this important publication will also incorporate data on the progress and challenges in reducing knowlesi malaria in regions where transmission occurs.

## Background

The recently released 2016 World Malaria Report, published by the World Health Organization (WHO), documents substantial progress towards control and elimination of malaria [1]. Malaria incidence rate has declined by 41% since 2000, and 21% since 2010. Mortality has fallen by 61% since 2000, and 29% since 2015. Seventeen countries eliminated malaria between 2000 and 2015, with a further 13 countries “approaching elimination.” These data represent significant achievements; however, major challenges remain. Challenges discussed in the World Malaria Report include the development and spread of mosquito resistance to insecticides, resistance to artemisinins and their partner drugs, and funding shortfalls. In Southeast Asia the emerging zoonotic malaria species *Plasmodium knowlesi* is an additional challenge that warrants regular inclusion in the World Malaria Report.

## Main text

*Plasmodium knowlesi* was reported as a major cause of human malaria in Sarawak, Malaysia, in a seminal paper published 13 years ago [2], and it is now the most common cause of malaria in this country [3, 4]. Cases have been increasingly reported elsewhere in Southeast Asia, including Indonesia, Thailand, Vietnam, and Myanmar, and in returned travellers [5, 6]. In the west of Indonesia, a country with an estimated 1.3 million malaria cases in 2015 [1], emerging evidence suggests that at least in some districts, *P. knowlesi* may now be the predominant *Plasmodium* species [7, 8]. In these regions limited evidence suggests that nearly all *P. knowlesi* cases are diagnosed by microscopy as *Plasmodium falciparum* or *Plasmodium vivax* [7, 8]. Thus, under-diagnosis of *P. knowlesi* may be common and incidence may be significantly underestimated across affected regions.

While mentioned in some previous editions of the World Malaria Report, *P. knowlesi* is not acknowledged in the 2016 Report. Regular inclusion of knowlesi malaria in the World Malaria Report, and acknowledgment of the importance of *P. knowlesi* as a cause of human malaria in parts of Southeast Asia, will facilitate efforts to increase recognition and improve surveillance of this emerging parasite.

*Plasmodium knowlesi* is a highly pathogenic malaria parasite in humans, with a low pyrogenic threshold and a risk of severe disease that in adults appears at least as high as that of *P. falciparum* [9]. In Malaysia, prior to widespread recognition of *P. knowlesi*, misdiagnosis of *P. knowlesi* was associated with delayed administration of parenteral therapy, and high fatality rates were reported [10, 11]. Increased recognition of *P. knowlesi* has led to more timely delivery of optimal treatment and has likely contributed to the reported sixfold reduction in *P. knowlesi* case-fatality rates [12].

In the 2016 World Malaria Report, Malaysia is listed as one of 13 countries approaching elimination. Elimination is defined as the interruption of local transmission of a specified malaria parasite in a defined geographic area, and in Malaysia, efforts have focussed on *P. vivax* and *P. falciparum* [13]. Indeed, Malaysia has made substantial progress in controlling these species [3, 14], and appears on track to approach elimination of human-only species by 2020. However, efforts to eliminate *P. vivax* and *P. falciparum* should not preclude efforts to control *P. knowlesi*. The WHO Malaria Policy Advisory Committee (MPAC) recognized the regional importance of this species by endorsing the creation of an Evidence Review Group on *P. knowlesi*, to address key gaps in knowledge and to “advise a path to malaria elimination that includes *P. knowlesi”* and to develop “appropriate mitigating and preventative strategies” [15]. Such strategies are likely to differ from those used for control of *P. vivax* and *P. falciparum*, and further research to increase recognition and identify effective interventions to limit transmission of *P. knowlesi* is required. The authors welcome the recent convening of the WHO Expert Consultation Meeting on knowlesi malaria, at which these issues were discussed.

## Conclusions

The role of the World Malaria Report is to educate and inform, to document progress and identify future challenges for controlling and eliminating malaria. While it is appropriate to focus on falciparum and vivax malaria, the major causes of global mortality and morbidity, the authors hope that in future years this important report will also incorporate data on progress and challenges in controlling knowlesi malaria in those regions where transmission occurs.
